# A systematic review of the intercontinental movement of unregulated African meat imports into and through European border checkpoints

**DOI:** 10.1016/j.onehlt.2023.100599

**Published:** 2023-07-20

**Authors:** S. Morrison-Lanjouw, R. Spijker, L. Mughini-Gras, R.A. Coutinho, A.L. Chaber, M. Leeflang

**Affiliations:** a University Medical Center Utrecht (UMCU/Julius Center), Utrecht, The Netherlands; b Cochrane Netherlands, Julius Center for Health Sciences and Primary Care, University Medical Center Utrecht, Utrecht University, Amsterdam Public Health, Utrecht, The Netherlands; c Netherlands Medical Library, Amsterdam UMC, University of Amsterdam, Amsterdam Public Health, Amsterdam, The Netherlands; d Centre for Infectious Disease Control (CIb), National Institute for Public Health and the Environment (RIVM), Bilthoven, The Netherlands; e PharmAccess Foundation, Amsterdam, The Netherlands; f School of Animal and Veterinary Sciences, The University of Adelaide, Adelaide, Australia; g Department of Epidemiology and Data Science, Amsterdam Public Health, Amsterdam University Medical Centers, University of Amsterdam, Amsterdam, The Netherlands; h Institute for Risk Assessment Sciences (IRAS), Utrecht University, Utrecht, The Netherlands

**Keywords:** Systematic review, One health, Bushmeat, Pathogens, Imports, Africa, Zoonotic disease, Customs, Species, Border, Biosurveillance

## Abstract

There is an urgent need for biosurveillance of unregulated African meat imports at border points of entry in destination markets. This is underscored by recent pandemics linked to exotic wildlife products. Our objective was to catalog the quantity of meat that is informally transported from Africa into and through Europe often without any veterinary or sanitary checks. We searched and included peer-reviewed studies that contained data on the intercontinental movement of unregulated meat from the African continent. This was followed by an investigation of the reported contamination of such meat. We included fifteen airport studies with limited data on this topic. The references included in this review describe the quantity of meat found at border inspection posts and the presence of pathogens. Disease-causing pathogens were found to be present, and the results are organized into bacteria, virus, and parasite categories. The species of animal meat found in this review were linked to CITES-protected species some of which are known reservoir hosts for infectious diseases. This represents a potential and unquantified human health risk to populations along the supply chain, and a loss to biodiversity in supply countries. Meat samples described in this review were primarily found opportunistically by Customs officials, indicating that any estimate of the total quantities passing undetected through border checkpoints must remain tentative, and cannot rule out the possibility that it is indeed considerably higher. We propose a template for future studies regarding African meat imports at border points of entry. The result of this review illustrates a gap in knowledge and lacunae regarding the amount of unregulated African meat imports worldwide, the pathogens it may contain, and the resulting biodiversity loss that occurs from the intercontinental movement of this meat.

## 1 Introduction

The most devastating pandemics have been caused by zoonotic diseases that include the Black Death, Spanish Flu, HIV/AIDS, SARS, MERS, and more recently COVID-19 [1]. Urbanization and globalization have now connected national and international rural and urban trade networks, facilitated by an increase in air travel. Three out of every four emerging infectious diseases are zoonotic in nature and often linked to exotic species found along supply chains in the wildlife trade [2]The growing list of emerging and reemerging infectious zoonotic diseases in the 21st century includes diseases originating from biosurveillance hotspots in parts of Africa known to have a significant demand for African wild meat (also referred to as “bushmeat”) [3]. Some diseases linked to African wild meat, such as Ebola, have been the cause of spillover events in remote areas in the past but have more recently furthered their spread as ecosystems are disturbed by anthropogenic activities and societal changes [4,5]. Pending the recognition and response to the animal reservoirs that lie at the animal, environmental and human interface, viruses, bacteria, and parasites are spreading throughout the global community through both the formal and informal trade of wildlife animal products.

Historical designations of specific hosts for a given infectious disease may no longer hold. Increased interaction between animal and human reservoir hosts as a result of ecosystem disturbances and climate change are also increasing the likelihood of a spillover event [6]. This, coupled with the fact that many African countries has limited capacity for morbidity surveillance, especially in isolated areas where wild meat is more often consumed, can impede the early detection along trade routes of what can quickly become a global threat to public health.

For large swaths of the West and Central African population, wild meat is consumed as an important source of protein and micronutrients [[7], [8], [9]]. This meat has been linked to emerging infectious disease outbreaks including the Ebola virus, Simian foamy virus, monkeypox virus, herpesviruses, retroviruses, Anthrax, and paramyxoviruses. With the spread of the African diaspora, so too has spread the custom of wild meat consumption and, accordingly, the potential reach of any accompanying pathogens. Today it is believed that the trade in African wild meat (also referred to as “bushmeat”) represents a multi-million-dollar trade with wide-ranging socio-political and health impacts [[10], [11], [12]]. While there is data on the diseases found along African wild meat trade chains on the African continent, information on the intercontinental movement of this meat, and the diseases it may carry, is lacking.

Recent Monkeypox outbreaks, outside of Africa and historically linked to African wild meat consumption, have resulted in an increased effort by the scientific community to identify as many transmission pathways as possible. As travel and population growth creates more opportunities for human and animal interactions, they are likely to increase the incidence of future global pandemics [13,14]. The demand for African meat amongst the African diaspora is largely unquantified but includes exotic species, some of which are linked to emerging and re-emerging infectious diseases Several studies have attempted to describe the demand for meat amongst the diaspora, but the underlying drivers of this demand have thus far gone underexplored [15,16]. More studies that track emerging infectious diseases along migration and trade routes are urgently needed. Although pandemics have historically traveled along trade routes, it remains a challenge to predict and prevent where the next pandemic may originate [17] [18]. This places global biosurveillance research at the center of pandemic prevention and preparedness.

To understand the key characteristics of the intercontinental movement of African meat, this study aims to assemble, and review published data on the quantity of unregulated African meat imports, as well as the range of associated species, including some known to be reservoirs of disease. Moreover, the conservation status of the animal species found, the pathogens detected in the meat products, and the country of origin of confiscated meat products at border points of entry were also summarized for each of the reviewed studies. Finally, known diseases associated with pathogens detected in this review are listed. The methodological quality of each of the included studies was then evaluated to formulate a template for the standardization of future studies in this domain. As a final output, a list of key components to include in future research was provided to standardize the methods used, maximizing comparability between study results.

## 2 Methods

### 2.1 Literature search and selection

We searched EMBASE, Medline, SCOPUS, and Web of Science in October 2019 and updated this search twice in February 2021 and November 2022 to capture any new relevant references. There was no language limitation. Search terms were organized into three key concepts; 1) African meat trade 2) global health and 3) biosurveillance and were used in various combinations including foodborne disease, food safety, air travel, bushmeat, African meat, airport, border, Customs, and national security.

Keywords included descriptions of African meat (domestic and wild), global health (i.e., one health), pathogens, disease, trade, travel, and customs. The search strings were developed and validated by the authors, drawing on their expertise in veterinary epidemiology, natural resource management, and the epidemiology of infectious diseases (Annex 1). A snowball approach was applied to all references listed in those academic papers that met all inclusion criteria to ensure that no relevant work was overlooked

The search for relevant studies was conducted in two phases. In Phase I we searched Web of Science, PubMed, and Google Scholar, using a combination of bushmeat, trade, Customs, airport, and imports terminology as part of our search criteria. The comprehensive search yielded 1860 results, excluding duplicates. Cases that failed to meet our criteria were first excluded after screening the titles of the references. After this process 1533 results remained. In Phase II, search terms were progressively added to help reduce the number of irrelevant cases and the remaining 30 full-text references were screened for inclusion criteria.

A total of 14 references remained after all the exclusion criteria had been applied and all duplicates removed (Annex 1). One ongoing study with study results shared by the Belgian government was added for a total of 15 references included in this review. The unpublished study was carried out in Brussels, Belgium, at the Zaventem International Airport, and included pathogen testing of African meat intercepted at Customs and describes the quantity of meat and species found. Preliminary findings for this ongoing study were presented at a sustainable wildlife trade conference in Brussels, Belgium [19]. However, because of the unpublished status of this study, we were able to include its preliminary results but could not assess the methodology used.

A limitation of the search criteria was that some studies yielded multiple publications, each in accordance with a (slightly) different research objective. In some cases, there was no in-text reference to precisely identify which African meat samples were used to investigate and publish alternate scientific objectives, increasing the risk of double counting when quantifying the overall number of animal samples found in this review. This is unlikely to have affected the overall objective of the study, which was to demonstrate a paucity in the academic literature that systematically quantifies and qualifies unregulated meat imports into destination markets.

The following criteria were used to determine which studies were viable for inclusion:

1. Studies must contain information on the topics of interest to our research. We selected studies only if they provided data for one or several of the following areas: quantity (of African meat imports into destination markets, the quality (i.e., presence of viruses, bacteria, and or parasites) of African meat imports, or both the quantity and quality of African meat imports into destination markets worldwide -regardless of whether African meat imports was the primary objective of the article. This required additional screening when the references were related to our topic because the data was sometimes deeply embedded in the results for another outcome of interest for a researcher.
2. Studies that did not report on data on African meat found, the quality of meat, pathogens found, and or the animal species of the meat, were excluded. Fish was not included in this review.
3. Only studies published between 2004 and 2023 (< 20 years) were included to ensure a reasonable cross-section of the available data on African meat confiscated at border points of entry into destination markets.

The review followed an iterative process; search terms were updated as the literature review progressed. New insights were derived from relevant conferences related to the wildlife trade and the intercontinental movement of African meat. Citations from relevant articles were scanned for missed case studies. While all border points of entry were included in the search protocols, there were no published studies focusing on port, air cargo, courier or land transport of unregulated African meat imported into destination markets worldwide. There were also no limitations imposed regarding the countries in which the studies were carried out.

As an additional step, we determined the conservation status of the various species seized at border points of entry. To do so, we first classified all included meat samples according to their taxonomic order. We then cross-referenced each animal entry with either the Convention on International Trade in Endangered Species of Wild Fauna and Flora (CITES) database, entering both the common name and scientific name to establish the presence of any trade protections for each respective animal. If no information was found, we subsequently checked the IUCN Red List as an additional measure of conservation risk. This step was included to provide some indication of the potential environmental damage being caused by the intercontinental movement of unregulated African meat. This paper makes no attempt to evaluate the efficacy of CITES trade restrictions.

### 2.2 Data extraction

Two independent readers (SJ and ML) screened all abstracts using the Rayyan online reference manager and retained only relevant entries. The remaining references were read through completely and independently and the decision to retain or exclude references was based solely on the previously discussed inclusion and exclusion criteria.

Upon selection of references that met inclusion criteria the relevant data was extracted and organized into a structured database using MS Excel. Each of the studies was recorded in the database according to author, study type, affiliation (i.e., academic/governmental), the primary purpose of the study, country of entry, name of the border point of entry, the total number of African meat seizures, questionnaires administered (Y/N), species detection (Y/N), methods of species detection, methods of storing samples, pathogens reported (% of seizures), direct/indirect flights, methods of unregulated import detection, African countries included in the study and the characteristics of the confiscated meat (i.e., boiled, raw, smoked, dry).

Since the purpose of this study was to evaluate the current state of peer reviewed academic research on unregulated African meat imports, import seizure data made available in grey literatures was not included. This review only generated one peer-reviewed reference that used retrospective data collected from an annual report from customs, at the airport under study. Additionally, notes, letters, and background articles were omitted.

### 2.3 Risk of bias assessment

We assessed to what extent the estimates of the amount of meat, species, or pathogens found, may have been biased. At present, there are no risk of bias tools to evaluate border studies on African meat imports. We, therefore, developed our own risk of bias checklist by assessing the included studies for deviations from a hypothetical ideal study design. This optimal design is reflected in a template we propose for future research.

In our methodological evaluation, we considered the risk of six possible forms of bias. In the context of an airport study of this kind, selection bias may occur in passenger profiling in which selected passengers receive extra screening above and beyond what all passengers undergo. The risk of selection bias can be minimized by implementing either a 100% security check of all passengers, or a properly randomized check. If the purpose, intervention, minimum sample size, study population, or outcome were not made clear, a risk of study design bias was indicated in the risk of bias table. Observer bias was evaluated by how data was collected by researchers and Customs officials who may have prior knowledge and subjective feelings about the study group and expected outcomes. Measurement bias was considered when all available measurement tools were not employed. This includes the use of x-rays, and dogs, a 100% baggage check of passengers on selected flights, the administration of questionnaires, and the consideration of temporal variations (i.e., hunting seasons) when planning the study period.

We also looked at the possibility of respondent bias for studies that either interviewed or administered questionnaires to passengers found to be carrying African meat in their luggage. For this review, respondent bias was defined as the possibility that people adapt their answers to avoid fines or modify their answers to adjust to the expectations of the researcher or Customs official. Reporting bias may be caused by the selective revelation of information, in retrospective airport seizure data used in some studies, which may cause over- or under-sampling or misjudgment of subjective measurements.

The final potential area for bias that we evaluated was the risk of misidentifying confiscated meat. This has significant implications for a proper risk assessment. The methods of species identification were varied and included visual identification, skeletal examinations, molecular determination (metabarcoding) and phylogenetic analysis. Visual identification of meat products represents a high risk of mischaracterization as a result of the systematic bias that occurs when customs agents and researchers are not trained to properly identify animal species. The risk of bias was divided into three categories: low, moderate, and high risk. A fourth category was added for studies that could not be classified due to insufficient information provided. We analyzed and synthesized the studies' findings by, first, documenting the range (quantity) of unregulated meat imports found at the border of destination markets.

The limitations of this evaluation included the difficulty in evaluating reporting bias when the primary research objective was not to detect African meat, but the reference was included because African meat was included in the study results of an included reference. Measurement bias is noted when all available methods are not employed, but the feasibility of the optimal design may vary as a function of financial constraints, government support or a possible lack of cooperation by Customs. Evaluating the feasibility was beyond the scope of this review.

### 2.4 Data analysis

We created an aggregate list of identified species of unregulated African meat. The list was then cross-referenced with the pathogens found and linked to known reservoir hosts to evaluate the potential public health risks associated with trade in the respective species (Table 2). All data were analyzed in a qualitative manner, as small sample sizes and varied reporting methods precluded any attempts at statistical analysis. After reviewing the main characteristics of the included references, a study design template was proposed to achieve maximum comparability between study results in the future (Annex 1).

## 3 Results

### 3.1 Risk of bias in reviewed studies

The risk of measurement bias was determined to be the most common source of potential bias for a wide range of different intervention methods. It should be noted that the primary objective of most included studies was not to find African meat imports therefore this evaluation should not be considered an evaluation of the study itself, but rather what criteria would be missing if the primary objective were to be the detection and description of unregulated African meat imports. Available detection techniques include detection dogs, x-ray imaging equipment and manual spot checks, a questionnaire for passengers found to be carrying unregulated African meat products, and a case-control study design to limit selection bias. The study that was retrospective using database search records or annual reports is particularly vulnerable to incomplete outcomes and methodological bias. In such cases, information regarding the passenger selection process and the luggage examination methods is frequently absent (Table 1**).**

**Table 1:** Overview of risk of bias in the reviewed studies.

Reference	Misclassification bias (animal species)	Selection bias	Design bias	Observer bias	Measurement bias	Respondent bias
[33]	N/A	Unknown	Low	Moderate	High	Unknown
[34]	Moderate	Unknown	Low	Moderate	High	Moderate
[35]	N/A	Unknown	Low	Moderate	High	Unknown
[36]	High	Low	Low	Moderate	High	Moderate
[37]	High	Low	Low	Moderate	High	Unknown
[38]	N/A	Moderate	Low	Low	High	Unknown
[21]	High	Low	Low	Moderate	High	Low
[20]	N/A	Unknown	Low	Low	High	Unknown
[39]	N/A	Unknown	Moderate	Unknown	High	Unknown
[40]	N/A	Unknown	Moderate	Unknown	High	Unknown
[41]	N/A	Unknown	Moderate	Unknown	High	Unknown
[42]	High	Moderate	Moderate	Low	High	Low
[43]	Low	Moderate	Moderate	Moderate	High	Unknown
[22]	High	Moderate	Low	Moderate	High	Unknown

There was generally a lower risk of bias in the other categories, though frequently there was insufficient information included for a proper evaluation. In the case of selection bias, half of the references did not report how passengers were selected for extra screening. Only two references report carrying out either a randomized or 100% security check of all passengers and describe how high-risk flights were determined. Respondent bias was largely unknown because if passengers were interviewed the methods were often not described. Only two references mention the use of questionnaires. Risk of observer bias was determined to be low to moderate in those studies which provided information for evaluation. Finally design bias was found to be generally low as most studies were clear about their research objective, sample size, target population and findings.

Only six references included descriptions of how species of meat samples found at border points of entry were identified. Reported methods included visual identification, skeletal identification, and phylogenetic analysis. Of these, visual identification carried the highest risk of bias, skeletal identification was considered moderately risky due to its inability to identify processed meat, and phylogenetic analysis was considered most effective.

### 3.2 Quantity of unregulated African meat imports

African meat entering destination markets was primarily found opportunistically by researchers and or Customs agents at border inspection posts. The lack of systematic search and seizure protocols across studies may have resulted in either the over- or underestimation of the quantity of African meat flowing through border points of entry, as well as the prevalence of the pathogens present in the meat.

As evident in Table 2, there was significant heterogeneity in reporting of quantities of African meat imports across studies. Of the references that reported the number of items confiscated, samples intercepted ranged between 3 and 168. Two references listed kilograms (Kg) as a measurement for the quantity of meat confiscated by Customs officials. This ranged between 150 to >200 kg. The number of confiscated samples identified as African wild meat, found per study, ranged from 9 to 44 items. The reporting of the amounts and descriptions of meat was not consistent across studies with some studies reporting both African domestic and wild meat for a total combined weight.

**Table 2:** Characteristics of the quantity of unregulated African meat imports.

Refererence	Destination country where African meat was Confiscated	Primary research goal: African meat (Y/N)	No. of African meat items seized (bushmeat/domestic) or weight (kilos)	Study Period (days/months)/location	100% security check	Country/countries of Origin	Description of meat
[33]		No	4 items (unspecified)	10 (Frankfurt)	No	South Africa	Dried game meat and sausage
	Belgium			110 (Berlin)			
[34]	France	Yes	33 items (domestic)	17 days (Paris)	Yes	Angola, Benin, Burkina Faso, Cameroon, Central African Republic, Chad, DRC, Republic of Congo, Guinea, Ivory Coast, Mali, Niger, Senegal,	Smoked, dried and raw
			9 items (bushmeat)				
[35]	France	⁎⁎Yes	⁎⁎18 items(bushmeat)	⁎⁎17 days (Paris)	Yes	West and Central Africa	Smoked, dried and raw
[19]	Belgium	Yes	61 items (mix of bushmeat and domestic meat)	January 2017–October 2018 (< 10 interventions)	Yes	Angola, Benin, Burkina Faso, Cameroon, Chad, DRC, Equatorial Guinea, Egypt, Ethiopia, Gabon, Guinea, Ivory Coast, Liberia, Mali, Morocco, Nigeria, Rwanda, South Africa, Senegal, Uganda	Smoked, dried, and raw
[36]	Brazil	No	16 items (PCIAR)	⁎6 days (Brazil Guarulhos (GRU))	No	South Africa, Angola, Egypt	Not reported
				6 days (Brazil Galeão (GIG))			
[37]	Brazil	No	Unknown	⁎6 days (Brazil Guarulhos (GRU)) 6 days (Brazil Galeão (GIG))	No	South Africa, Angola, Egypt, Morocco	Not reported
[38]	Brazil	No	210 k (Nigeria)		No	Angola, Cameroon, Cape Verde, Chad, Congo, Egypt, Ghana, Ivory Coast, Morocco, Mozambique, Uganda, Kenya, Libya, Namibia, Niger Nigeria, Senegal, South Africa, Sudan, Tunisia, Zambia, Zimbabwe	Not reported
			209 k (South Africa)	⁎⁎⁎Database study/Retrospective design			
			101 k (Angola)	2006–2009			
[21]	Switzerland	Yes	168 items				
	⁎⁎⁎⁎1140 days (Zurich)	No	West, Central, East, and South Africa, Benin, Cameroon, Ivory Coast and Ghana	Dried, canned, smoked, boiled, salted, grilled, fried and frozen			
			22 items (bushmeat)	⁎⁎⁎⁎1260 (Geneva)			
[20]		No	∼ 150 k (2010)	Study Period from 2014 to 2018	No	North, Central and S. Africa	Not reported
	Germany		∼ 150–200 k (2012)	**NCDU**			
			200+ kilos (2014)	1A (Eastern Germany International Airport)			
				Airport 1B (Central Germany)			
[39]		No	3 items	Total study period = 406 days	No		Not reported
	Spain			Bilbao		Morocco (N. Africa), Equatorial Guinea (Central)	
				**NCDU**			
[40]		No	3 items	Total study period = 406 days	No	Morocco (N. Africa), Equatorial Guinea (Central)	Not reported
	Spain			Bilbao			
				**NCDU**			
[41]		No	3 items	Total study period = 406 days	No	Morocco (N. Africa), Equatorial Guinea (Central)	Not reported
	Spain			Bilbao			
				**NCDU**			
[42]	Austria	No	3 items	Total number of days = 206	No	Nigeria, South Africa, Ethiopia	Dried and cured
				**NCDU**			
[43]	United States	Yes	44 bushmeat items (Customs)	Study period October 2008–September 2010	No	Nigeria, Liberia, Guinea	Raw, lightly smoked, dried
			8 bushmeat items (postal shipments)	5 International airports: NY, DC, PA, TX, GA			Most items contained moist inner tissue
			20 bushmeat items (USFWS)				
[22]	France	Yes	4 items	Study period January 2017–October 2018	No	Central Africa	Smoked

USFWS- United States Fish and Wildlife Service.

PCIAR – People identified as carrying illegal animal products.

NCDU- Number of actual confiscation days unknown.

⁎ Same study used for different outcomes (i.e., the detection of a pathogen(s)).

⁎⁎ where indicated some data was used in multiple articles written by the same author answering different research questions.

⁎⁎⁎ Assuming that samples taken correspond 1:1 with confiscated samples.

⁎⁎⁎⁎ Unknown number of study days (data from database search).

**Fig. 1:**
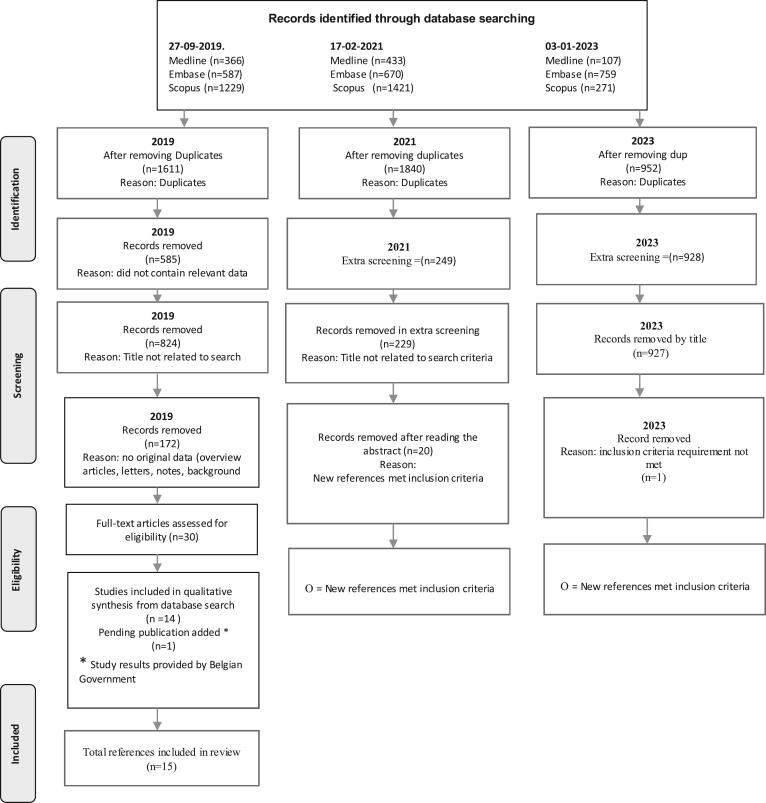
Flow chart of the literature search and study selection process based on the PRISMA 2020 standard for systematic review. (see attached file)

Only three studies used a standardized method to detect meat at border inspection posts. The methods used were either a 100% security check which involves the manual checking of all passenger luggage occasionally accompanied by X-ray imaging, or a targeted Customs search based on their own risk analysis. Of the fifteen references included in this review, multiple references were generated from the same underlying study (Annex 1). As illustrated in Fig. 1, Fig. 3 references were from Spain (one study), 3 from France (two studies), 3 from Brazil (one study), 1 from Belgium (one unpublished), 2 from Germany, one from the United States (US), one from Switzerland and one from Austria. Most studies that include data on unregulated African meat imports were conducted in Europe followed by Brazil and the US.

### 3.3 Microbial quality of the confiscated meat

This review reveals certain characteristics that may affect the ability of pathogens to survive the trip from Africa into and through Europe, North America, and Brazil. Eight references reported these characteristics. The unpublished Belgian study describes that long storage periods, where leaking luggage was kept in airport freezers for extended periods of time prior to analysis, may have resulted in the loss of most viruses before pathogen testing could be carried out. Seven references described that dried African meat was found and confiscated. Six of the studies found smoked meat and 50 % found raw, uncooked meat. (As Customs officials frequently found more than one method of meat preservation, these values do not add up to 100%). Other descriptions of the meat found included canned, boiled, salted, or cured meat, and meat in the form of sausages. One reference described that some confiscated meat samples contained moist inner tissue. Raw and meat containing an uncooked center represent a heightened risk of pathogens making the trip over from Africa to destination markets.

Half of the references included in this review reported following International Organization for Standardization (ISO) guidelines when collecting and testing samples for pathogens in their study. Of these, six specified which guidelines were used. The most commonly used pathogen testing technique for identification was polymerase chain reaction (PCR), though metagenomics for viruses and parasites and rRNA gene metagenetic analysis for bacteria was also described.

The six most prevalent bacteria identified in this review (in descending order) were *Staphylococcus aureus, Listeria, E.Coli, Brucella, Salmonella,* and *Enterococcus* though these may not be found in the most commonly identified animals. Viruses detected include *Herpesviruses, Simian Foamy Virus, Poxviridae, retroviridae* and *papillomaviruses, Hep E and African swine flu (ASF).* The complete list of detected pathogens can be found in Table 3.

**Table 3:** Pathogens found in the meat analyzed in the reviewed studies.

Reference	Viruses	Bacteria	Parasites
Beutlich et al. (2015) [33]		*Brucella, Yersinia, Listeria, Salmonella*	
Chaber et al. (2016) [35]		*Listeria,Klebsiella, Citrobacter S., Streptococcus,Staphylococcus aureus*	
Chaber and Gagliany (2019) [19]	African Swine Flu	*Brucella, E. coli, Yersinia, S. aureus, Taylorella E., Shigella, Edwardsiella T., Ehrlichia ruminantium, Erysipelothrix rhusio, Dermatophilus congolensis, anaplasma, Bartonella hen., Coxiella Bur., Bacillus anthracillus*	*Babesia, Theileria*
Rodriguez-Lazaro et al. 2015a [39]		*Staphylococcusn aureus*	
Rodriguez-Lazaro et al. 2015b [40]		*Salmonella, Listeria*	
Rodriguez-Lazaro et al. 2015c [41]	Hep E.		
Schoder et al. 2015 [42]		*E. coli, Salmonella, Coagulase Staphyloccoci*	
Smith et al. 2012 [43]	Herpesviruses (including CMV: betaherpesvirus and LCV: gammaherpesvirus), SImian Foamy Virus		
Temmam et al. 2017 [22]	Herpesviruses, Papillomaviruses Poxviridae, Tymoviridae, retroviridae, Anelloviridae Mimiviridae, Siphoviridae, Myoviridae, Podoviridae, Phycodnaviridae and Ascoviridae	*Staphylococcus, Listeria, Enterococcus, Baccilus* sp.*, Lactobacillus* sp.*, Entercoccus sp, Cronobacter*	*Spirometra erinaceieuropaei, Haemonchus placei, Wuchereria placei (roundworm), Leishmania, Plasmodium, Schistosoma rod.*

### 3.4 Animal species of the confiscated meat

The most frequently identified African meat species was from the animal order of primates, which are known to be reservoir hosts for a range of both communicable and non-communicable diseases (Table 4). Eleven of the primates reported to be found in this review are protected by CITES with the first and second highest level of protection (CITES I/ II). The identification and cataloguing of animal species along trade routes is an important first step in the biosurveillance of both known and unknown natural reservoirs as well as intermediary hosts of disease. The top four taxonomic orders (from most to least commonly found) are: Primates, Artiodactyla, Rodentia and Pholidota (Table 4). Animals in the Squamata, Crocodylia, and Testudines were also reported (Tables 4) along with insects from the Hymenoptera order. These orders contain known reservoirs hosts of disease that include primates, duikers and pangolins [2].

**Table 4:** Animal species identified in the systematic review and CITES/IUCN conservation status. (see attached under bibliography).

Artiodactyla	Common name	Reference	CITES Appendices or IUCN Red List Status*
Antelope	Unspecified	[21,39]	CITES I/CITES II
*Philantomba monticola*	Blue Duiker	[19,34]	CITES II
*Cephalophus dorsalis*	Bay duiker	[19,34]	CITES II
Cephalophus spp.	Duiker	[19]	CITES II
Philamtomba walteri	Walter's duiker	[19]	CITES II
*Potamochoerus porcus*	Red River hog	[19,34]	IUCN LC**
*Redunca arundinum*	Southern Reedbuck	[19]	IUCN LC**
*Sylvicapra grimmia*	Common duiker	[19]	IUCN LC**/CITES II
*Tragelaphus eurycerus*	Bongo	[19]	CITES III
*Tragelaphus scriptus scriptus*	Cape Bushback	[19]	IUCN LC**
*Rodentia*			
*Atherurus africanus*	Brush-tailed porcupine		IUCN LC**
*Hystrix cristata*	Crested Porcupine	[34]	IUCN LC**
*Cricetomys gambianus*	Gambian pouched rat	[19]	IUCN LC**
Cricetomys sp. 3	African giant pouch rat	[19]	IUCN LC**
*Thryonomys swinderianus*	Greater Cane rat	[19,34]	IUCN LC**
Porcupines	Unspecfied	[21]	IUCN LC***
Rodents	Unspecified	[39,43]	IUCN LC***
Thryonomys sp.	Lesser Cane rat	[19]	IUCN LC***
*Xerus erythropus*	Striped ground squirrel	[19]	IUCN LC***
*Pholidota*			
Pangolin	unspecified	[21]	CITES I
*Manis tetradactyla*	Long-tailed pangolin	[34]	CITES I
Smutsia gigantea	Giant pangolin	[19]	CITES I
*Manis tricuspis*	Tree pangolin	[19]	CITES I
*Primates*			
*Chlorocebus sabaeus*	Green monkey	[23]	CITES II
Cercopithecus nicitans	Greater white-nosed monkey	[23]	CITES II
*Papio Papio*	Baboon	[23]	CITES II
*Cercocebus atys*	Sooty mangabey	[23]	CITES II
*Pan troglodytes ellioti*	Nigerian-Cameroonian chimpanzee	[23]	CITES II
Cercopithecidae	De Brazza's monkey	[24]	IUCN LC **
Cercopithecidae	Greater spot-nosed monkey	[19]	CITES II
Cercopithecidae	Savannah monkey	[19]	IUCN LC **
Cercopithecidae	Grivet monkey	[19]	CITES II
Cercopithecidae	Guenon monkey	[22]	CITES I/II***
Cercopithecidae	Collared mangabey monkey	[22]	IUCN Endangered/CITES II
Cercopithecidae	Mustached guenon monkey	[19]	CITES II
Cercopithecidae	Yellow baboon	[19]	CITES II

*CITES Appendix I- includes species threatened with extinction. Trade in specimens of these species is permitted only in exceptional circumstances, Appendix II includes species not necessarily threatened with extinction, but in which trade must be controlled to avoid utilization incompatible with their survival, Appendix III contains species that are protected in at least one country, which has asked other CITES Parties for assistance in controlling the trade.**Population numbers may be decreasing in the wild but considered least concerned (LC) in conservation efforts *** Likely to for this sample to fall into this conservation category.

Primates may pose an extra risk, given their genetic resemblance and evolutionary ties to humans. The studies we included found monkeys and primates that included apes, mangabey species, green monkeys, grivet monkeys, greater and lesser white-nosed monkeys, mustached monkeys, baboons, De Brazza's monkeys and four other unidentified samples from the Simian and Cercopithecidae families (Table 4). The most commonly identified species of Primates were baboons, grivet monkeys and mangabey species.

Ten animals were identified in the Artiodactyla order, of which the most common were bay and blue duikers along with red river hogs. The unidentified antelope is likely to belong to the *Cephalophus zebra*, *Hippotragus niger* or *Hippotragus niger variani* based on the countries of origin.

In the reported samples of the Rodentia Order, different varieties of rat were detected including the African giant pouched rat, Gambia pouched rat and the Greater and Lesser Cane rat. Porcupine was also reported. Four rodent samples were unidentified. The samples of Pholidota included the giant pangolin, the tree pangolin, the long-tailed pangolin. One pangolin sample was left unspecified. The meat of dwarf- and Nile crocodiles were also reported.

### 3.5 Methodological quality of the reviewed studies

The methods for detecting meat differed vastly between studies ranging from manual inspections to less invasive x-ray machine imaging. Eight studies conducted manual inspections in which luggage was opened and searched although it is unclear what protocols each study used to carry out the inspection. X-ray imagery was used in 5 studies and two studies indicated they used targeted profiling of either origin of flights and or passengers. In some studies, the selection of flights was informed by the previous experiences of Customs officials. Other studies used previous literature as a basis for their selection of flights. The Zaventem Airport study in Brussels, Belgium, also included targeted profiling where passengers were identified as having a higher probability of carrying products of animal origin from Africa [19].

Two studies described using X-ray technology and one study included the use of both X-ray technology and manual inspections. A further two studies reported only using a manual detection technique for luggage inspection while the inspection methods of remaining references were not reported. In the cases in which only manual inspections were carried out passengers were selected for extra screening based on the suspicions of Customs officers. When X-ray screening was used first, all suitcases were initially scanned and only opened if flagged as suspicious. It is unclear if any special training was provided to Customs officials to identify meat products of different types (raw, smoked, dried), which would likely affect detection rates.

It is unknown how much of the new seizure data generated by the EU studies in this review was included in the total amount of unregulated African meat seizures reported by EU customs to the European Commission pursuant to Annex VI of (EU) 206/2009. EU member states are asked to submit an annual report to the European Commission (EC) summarizing the relevant information about the measures implemented to enforce the rules laid down in regulation (EU) 206/2009, and about the results thereof (EC, 2009) [20]. However, it is unclear how many EU members include animal species when reporting on confiscated meat, and whether the data is complete.

Three studies reported the use of x-ray machines that resulted in the detection of unregulated African meat but only one study reported the efficiency of the x-ray machines used [21]. In this case, it was believed to be operating at between 85% and 100% efficiency. Unfortunately, as this was the only study in which such information was included, it is impossible to compare across references. There were only two studies conducted at smaller airports catering primarily to domestic flights, all other airports in this review were large international airports. Conducting studies at small, domestic-oriented airports complicates the process of identifying the country of origin of detected meat samples as it may not align with the flight origins [21, 22].

The heterogeneity of the methodologies implemented in the referenced studies presented a challenge when attempting to compare results. In the absence of baseline data, risk assessment agencies cannot detect trends. The lack of comparability imposed by methodological heterogeneity is a significant impediment to the compilation of a credible baseline. To this end we propose a universal template for future studies in this domain.

## 4 Discussion

Europe has been revisiting its strategy for securing the food safety of products, including wild game, since the turn of the century. European laws collectively referred to as hygiene regulations include (EC) nos. 178/2002, 852 to 854/2004, 882/2004, and 2075/2005 (EC, 2002, 2004 [23]. This set of laws underscores the importance of the traceability of all animal products throughout the food chain, to enable the withdrawal of meat products that do not meet hygiene and proper cooling standards for transportation. They do not extend to unregulated African meat imports into and through Europe.

This study describes the available data in the literature on the characteristics of unregulated African meat imports into destination markets worldwide. The findings underscore that systematic research is needed in this domain to improve pandemic prevention, as well as pandemic preparedness. The review reports on the quantity of African meat intercepted at Customs, the pathogens found when pathogen testing was performed, the species of animals concerned, as well as their conservation status of the included references.

The results of this review can, moreover, be linked to numerous UN sustainable development goals (SDGs), such as those that relate to poverty (SDG 1), hunger and food security (SDG 2) good health and well-being (SDG 3), quality education (SDG 4), responsible production and consumption (SDG 12), climate action (SDG 13) and life on land (SDG 15), which includes biodiversity loss [24]. This further emphasizes the fact that the intercontinental movement of unregulated African meat lies at the intersection of animal, human and environmental health and therefore can only be properly addressed using a One Health approach.

The first objective of this review was to provide a sense of the quantity of unregulated meat imports entering destination markets from biosurveillance hotspots in Africa [25]. Because of the broad range of quantities described in this review, it is likely that the meat may be intended for both individual use as well as for organized trade. Methods of detection described in this review are heterogeneous across border inspection posts with most meat products found opportunistically. A proper assessment of the magnitude of these imports is further complicated by the illegal nature of this activity, which disincentivizes passengers from revealing the true animal species that they are carrying into the country. Additionally, the use of transit flights and smaller airports by traffickers further complicate the process of mapping the true scale of the trade, as well as of tracing the complete path of the meat from origin to destination. These factors cast doubt on the estimated quantities of unregulated African meat imports, which may affect the validity of local risk assessments related to public health.

A paucity of data regarding the quantity of unregulated African meat entering countries may negatively impact such periodic risk assessments as the most recent European Food Safety Authority report on the risk of Ebola through African wild meat consumption [26]. The report questions the existence of unregulated African meat imports entering the EU and the ability of pathogens to survive the trip from Africa to destination cities around the world. The report also assumes a low demand for African wild meat in Europe, possibly resulting from the results of local ad-hoc Customs seizures and a lack of understanding of the drivers of local demand in destination markets. If this assumption is indeed inaccurate, then the resulting assessment and related policy agendas may not reflect reality.

This review suggests that the import of raw meat products and numerous meat samples containing moist inner tissue merits further investigation, as dried or smoked meats compared to uncooked or partially smoked meats likely represent different levels of health risks. Most African meat found by Customs is immediately incinerated pursuant to local biohazard protocols. It should be noted that this prevents the scientific community from identifying animal species and testing meat for pathogens along intercontinental supply chains.

The risk of bias (Table 2) also illustrates the heterogeneity and risk of bias in the methodologies used across studies that included data on unregulated African meat imports. A minority of references set out to investigate unregulated African meat imports as a primary objective of their study. Therefore, the risk of bias table serves less to evaluate a study's success in detecting African meat, and more to guide researchers in the design of a gold standard study for studies intending to further investigate the characteristics of the intercontinental movement of African meat. It should be noted that no information about funding restrictions, or the lack of government support in the form of permissions to carry out required activities, was available in the studies reviewed. As a result, study limitations conveyed in the risk of bias table may reflect a lack of funding, government administrative support and/or the potential obstacles presented by autonomous law enforcement entities.

**Fig. 2:**
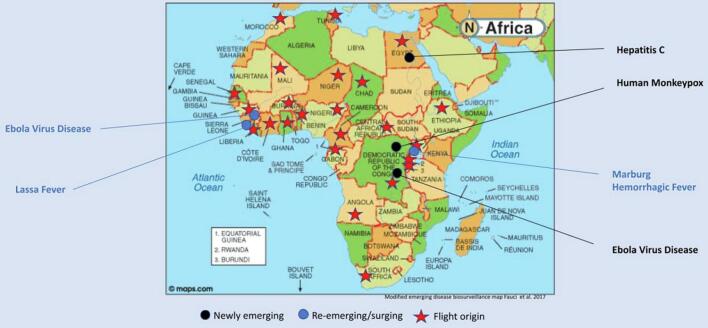
Map of the supply countries from which unregulated African meat originated, superimposed on a map of emerging and re-emerging infectious diseases in Africa (see attached file**).**

The second objective of this review was to describe the quality (i.e., pathogen load) and characteristics of the African meat found at border points of entry and the countries from which it originates. Numerous pathogens have been recognized as important disease-causing agents as they are subject to mutation and thus evolution which can create biosurveillance hotspots for emerging infectious diseases. As seen in Fig. 2, the country of origin of many flights containing unregulated African meat products are in close proximity to biosurveillance spots of numerous emerging and re-emerging infectious diseases. This illustrates that potentially contaminated meat is moving into and through Europe from areas that are predicted to have a high relative risk distribution of spillover events [27]. This is particularly concerning, given that the non-human primates, rodents, and bats identified in the meat samples included in this review have been identified as potential sources of spillover of zoonotic diseases into humans [2,6]. This poses significant risks to animal and human health in both supply and demand countries of the African meat trade, as well as an unquantified and unacknowledged occupational hazard to individuals such as baggage handlers and customs officials who may come into contact with contaminated meat on a regular basis.

**Fig. 3:**
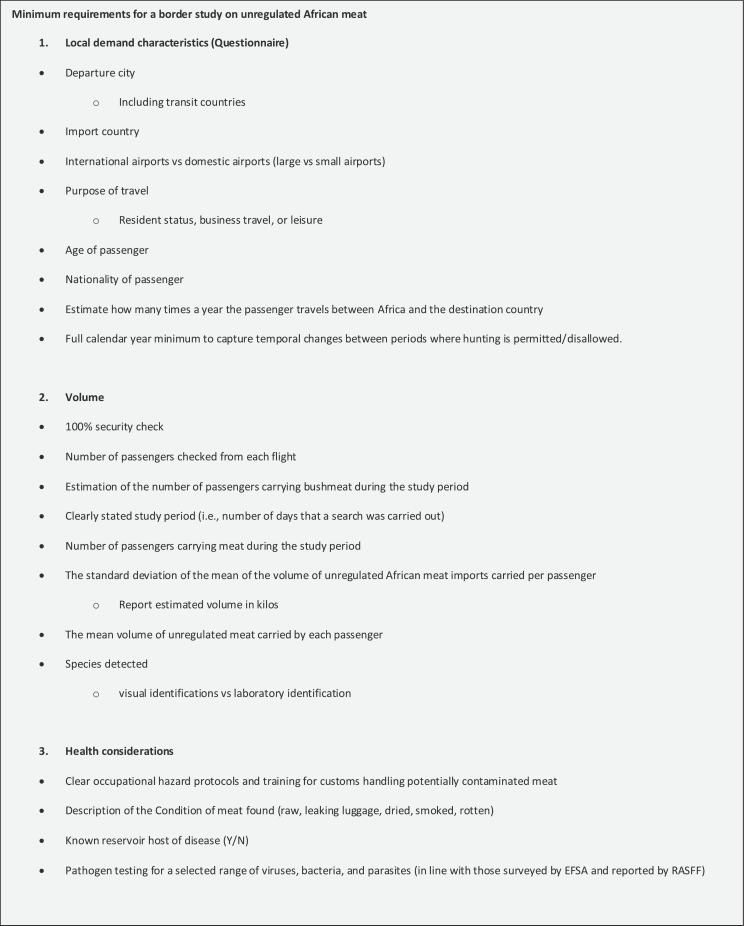
Template for future studies (see attached file).

There is a growing body of knowledge of bacterial pathogens found in bushmeat and wet markets worldwide. Examples include *Staphylococcus aureus, Listeria, E. coli, Brucella, Salmonella, Enterococcus, Shigella* and *Campylobacter* spp., consistent with the pathogens found at border points of entry included in this review [[28], [29], [30]]. Some of these pathogens, such as Listeria, are considered a high-priority public health risk by the European Rapid Alert System for Food and Feed (RASFF), which is an important tool for supranational communication of food safety risks along global supply chains of *regulated* meat [31]. The viruses included in this review include African swine fever (ASV), cytomegalovirus, lymphocryptovirus, simian foamy virus, and Herpesviruses. The potential for future and continued spillovers from Simian foamy viruses from such species found in this review as the green monkey is estimated to be significant by a growing body of literature [2].

Detecting the quality of unregulated meat that moves along trade and transit routes was complicated, in some studies, by the discovery of falsified health certificates for livestock meat products and especially fresh meat. While this is an important aspect of measuring the quality of meat imports, it was only mentioned in two of the references included in this review.

This review attempts to aggregate and evaluate the current body of research on the breadth and characteristics of the unregulated African meat trade, however it should be noted that there are some factors that may confound our efforts. One such issue is the lack of reporting of non-results. In this review, the listing of pathogens for which tests were carried out but no pathogens were found occurred only exceptionally, which complicates any attempt to assess the risks associated with this trade. Most significant is the small sample size of studies that met all the inclusion criteria. While this is indicative of the relatively unexplored nature of this trade, such small samples can only indicate the presence, not prevalence, of pathogens found in unregulated meat imports. Additionally, some studies that detected a taxonomic assignation of reads for pathogens were not able to confidently attribute these to a family of viruses or other micro-organisms. In this case, they corresponded to repeated patterns when mapping against reference genomes. This may have resulted in the exclusion of pathogens reported and from our final list of pathogens detected.

The potential economic fallout from the entry of problematic pathogens is highlighted by examples such as African swine flu and COVID-19. Some experts predict that the full cost of the COVID-19 pandemic may reach $ 10 trillion in forgone Gross Domestic Product (GDP) over 2020–21, significantly hindering progress toward numerous sustainable development goals [32]. The presence of pathogens found in confiscated meat products may also represent a poorly understood risk to local agricultural sectors, exemplified by the detection of the African swine fever. This example further underscores large economic costs and a threat to global food security that can be linked to several UN sustainable development goals [24].

## 5 Conclusions

The current knowledge gap described in this review may affect the validity of human and animal health risk assessments accepted by local governments. A One Health approach to simultaneously address animal, human, and environmental health, along the international commodity chain of unregulated African meat imports is warranted. This review was designed to provide a foundation upon which future research can begin to catalog what bacteria, viruses, and parasites are entering into and through Europe from Africa, via unregulated African meat imports. This analysis may be useful as competent authorities regularly revise disease transmission prevention protocols. In the absence of standardized and systemic biosurveillance as it relates to African meat imports, local government ministries may be operating with a biosurveillance blind spot. While many associated biosafety risks may be relatively minor, the potential exists that were they to occur they could have significant impacts on animal and human health. Harmonized surveillance of the importation of illegal meat, at European border points of entry, is warranted in support of pandemic prevention. The elevated conservation status of the species found in this review suggests that more education efforts and harsher penalties are needed to curb the handling, transportation, and consumption of illegal meat products into and through Europe.

## Declaration of Competing Interest

None.

## Figures and Tables

**Table:** 

Step 1. African Meat Trade	Step 2. Global Health	Step 3. Biosurveillance	Database search strings
International bushmeat African meat Air travel Passengers Customs Scale Trade Illegal Quality of bushmeat at airports Quantity of bushmeat at airports African meat imports Europe Smuggling International Bushmeat AND Trad*, Air travel, passenger*, African wild meat, African meat, meat from Africa, EU, Europe*, quantity AND bushmeat OR African meat, quality of African meat OR bushmeat AND EU OR Europe, “scale of bushmeat trade”, scale AND bushmeat or African meat, illegal imports AND bushmeat or African meat, smuggl*, animal food product confiscate*, border points of entry, Customs, cargo, freight, ports, airport*, air travel	Global health Transmission Pathogens Viruses Food Safety Foodborne disease Globalization Trade Transport Cross over species Bushmeat African wild meat Seizure Airport Confiscation Global health and Africa meat/bushmeat, bushmeat and Europe*, food safety AND bushmeat AND airport, transmission, pathogen* and bushmeat seizure, bushmeat AND disease prevalence, cross-over species risk AND Bushmeat or African meat AND imports or seizures OR confiscat*, bushmeat or African meat and viruses AND Transport or Travel or Air or Passenger*, zoonoses, zoonotic disease*, food safety	Biosecurity Biosurveillance Africa Meat Wild Bushmeat Product of Animal Origin Border Control Animal Disease National Security Biosurveillance and Africa* meat or bushmeat, food product* of animal origin AND Africa and airport or seizure or border control or Customs, animal disease*, foodborne disease, national security, border control*, biosecurity AND bushmeat OR African Meat or MEAT from Africa	**Embase** POAO.ti,ab. Meat.ti, ab,kw. Bushmeat.ti,ab. Simian-game.ti, ab. ((wildlife or primate or animal)adj2(origin or based or product))).ti,ab,kw. (zoonoses or zoonotic).ti, ab,kw.or exp. Zoonoses/ 1 or 2 or 3 or 4 or 5 or 6 (border or Customs or seized or (illegal* adj3 import* or confiscated).ti, ab,kw. 7 and 8 Limit 9 to (conference abstracts or embase **Medline** POAO.ti,ab. Meat.ti,ab,kf. bushmeat.ti.ab. simian-game.ti,ab. ((wildlife or primate or animal) adj2 (origin or based or product)).ti,ab,kf. (zoonoses or zoonotic).ti, ab,kf or exp. Zoonoses/ 1 or 2 or 3 or 4 or 5 or 6 (border or Customs or seized or (illegal*adj3 import*) or airport* or confiscated).ti,ab,kf. 7 and 8 **Scopus** (TITLE-ABS-KEY (poao OR meat OR bushmeat OR simian-game OR ((wildlife OR primate OR animal) W/2 (origin OR based OR product)) OR zoonoses OR zoonotic)) AND (TITLE-ABS-KEY ((border OR Customs OR seized OR (illegal* W/3 import*) OR airport* OR confiscated)))

## Data Availability

Data will be made available on request.
